# What Is the Nature of Poststroke Language Recovery and Reorganization?

**DOI:** 10.5402/2012/786872

**Published:** 2012-12-23

**Authors:** Swathi Kiran

**Affiliations:** ^1^Department of Speech, Language, and Hearing Sciences, Sargent College of Health & Rehabilitation Sciences, Boston University, 635 Commonwealth Avenue, Boston, MA 02215, USA; ^2^Massachusetts General Hospital, Boston, MA, USA

## Abstract

This review focuses on three main topics related to the nature of poststroke language recovery and reorganization. The first topic pertains to the nature of anatomical and physiological substrates in the infarcted hemisphere in poststroke aphasia, including the nature of the hemodynamic response in patients with poststroke aphasia, the nature of the peri-infarct tissue, and the neuronal plasticity potential in the infarcted hemisphere. The second section of the paper reviews the current neuroimaging evidence for language recovery in the acute, subacute, and chronic stages of recovery. The third and final section examines changes in connectivity as a function of recovery in poststroke aphasia, specifically in terms of changes in white matter connectivity, changes in functional effective connectivity, and changes in resting state connectivity after stroke. While much progress has been made in our understanding of language recovery, more work needs to be done. Future studies will need to examine whether reorganization of language in poststroke aphasia corresponds to a tighter, more coherent, and efficient network of residual and new regions in the brain. Answering these questions will go a long way towards being able to predict which patients are likely to recover and may benefit from future rehabilitation.

## 1. Introduction

About 795,000 Americans each year suffer a new or recurrent stroke. That means, on average, a stroke occurs every 40 seconds. Aphasia (or the inability to communicate) is the effect of a stroke in the left (and sometimes right) hemisphere of the brain. Typically, damage to regions in the left perisylvian network, including the inferior frontal gyrus (IFG), middle frontal gyrus (MFG), angular gyrus (AG), supramarginal gyrus (SMG), superior temporal gyrus (STG), middle temporal gyrus (MTG), inferior temporal gyrus (ITG), and the supplementary motor area (SMA), results in aphasia. There is a large body of research evidence suggesting that the brain undergoes tremendous recovery and reorganization of structure and function following a stroke. That is, specific linguistic impairments, such as phonological disorders, lexical semantic impairments, and syntactic impairments, show substantial recovery in the first few months following a stroke. Hillis et al. [1, 2] suggest that recovery of language function after stroke occurs in three overlapping phases, each with a unique set of underlying neural phenomenon. The initial phase is called the acute phase and lasts for about 2 weeks after the onset of the lesion. The second phase is the subacute phase, and this usually lasts up to 6 months after onset. Finally, the chronic phase begins months to years after stroke, and it may continue for the remainder of the person's life.

Each of these phases is accompanied by a tremendous amount of physiological change but a lot is unknown about the precise mechanisms underlying language recovery in poststroke aphasia. There have been several recent reviews examining advances in neuroimaging of recovery from aphasia [3–5], and all of them underscore the need for further careful and systematic research in this topic. Moreover, even in situations when language is recovered, it is not known whether the regions of activation observed are truly due to reorganization of language abilities to other functionally capable regions or due to utilization of abnormal cognitive strategies. While much progress has been made with regards to methodological advances in measuring neural changes in poststroke aphasia and in terms of demonstrating stability of performance and reliability in brain activation regions across repeated fMRI sessions in aphasic patients [6–11], the mechanisms promoting recovery and reorganization of language in poststroke aphasia are still unresolved. It should be noted that this review does not address detailed methodological procedures or the associated challenges related to the use of fMRI in poststroke aphasia. The reader is referred to a recent set of papers that address issues that include selection of baseline tasks [12], structure of the experimental language tasks [12], mitigation of motion related artifacts [13], reliability and stability of fMRI images across sessions [13], analysis of fMRI data in stroke patients [13], analysis of lesioned images [14], and analysis of treatment experimental designs to measure brain plasticity [15].

In this paper, the first section explores the basic anatomical and physiological substrates of brain regions in poststroke aphasia, including the nature of the hemodynamic response in patients with poststroke aphasia, the nature of the peri-infarct tissue, and the neuronal changes in the peri-infarct tissue and ipsilesional hemisphere. Given the relative paucity of studies examining the mechanisms underlying language recovery in the infarcted hemisphere, this paper interleaves studies examining language recovery with studies examining motor recovery, for which we have a more advanced understanding [16–18]. The second section details the nature of language recovery in poststroke aphasia by reviewing studies that have examined language recovery in the acute phase, subacute phase, and the chronic phase. This section also reviews studies that have examined the nature of rehabilitation-induced plasticity in poststroke aphasia. The third and final section examined changes in connectivity as a function of recovery in poststroke aphasia, specifically in terms of changes in white matter connectivity, changes in functional effective connectivity, and changes in resting state connectivity in poststroke brains. Again, studies on connectivity studies with respect to language recovery are just emerging, whereas there are several reviews on changes in network connectivity related to recovery of motor limb function [19]. Thus, this section of the paper also integrates studies on language and motor recovery. Clearly, understanding the nature of plasticity in the motor cortex does not easily parallel the nature of language recovery; however, the methodology implemented to understand plasticity is of great value to the emerging literature on plasticity mechanisms underlying language recovery. The following are the questions asked in the study.

## 2. What Are the Basic Anatomical and Physiological Substrates of the Infarcted Hemisphere in Poststroke Aphasia?

**Figure 1:**
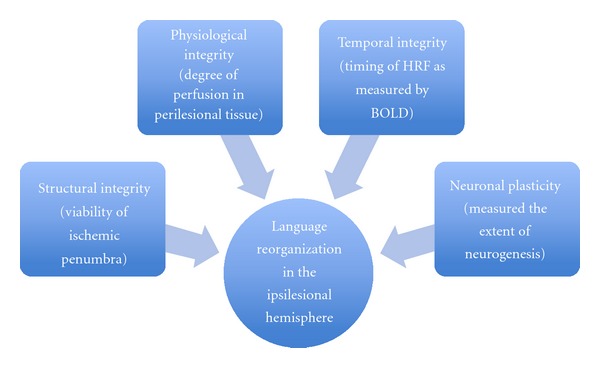
Schematic of factors that influence the potential of recovery language function in the infarcted hemisphere.

Stroke, or cerebral ischemia, has a clear neurological consequence, with disruption in blood supply causing infarction and ultimate necrosis of tissue. The effect of the infarction is an alteration of blood flow within the infarcted hemisphere and accompanied changes in the neuronal representation. In order for recovery of function to be restored to the infarcted hemisphere, its structural, functional, and physiological integrity will need to be at optimal operationality to sustain such recovery (see Figure 1). It is not clear, however, to what extent the infarcted hemisphere can sustain recovery of function as there are several factors that may influence its viability.

### 2.1. What Is the HDR Signal in Patients with CVA and Aphasia?

Blood oxygenation level-dependent (BOLD) functional MRI (fMRI) is the method that is most commonly used to examine the nature of brain function. A change in BOLD signal corresponds to a decrease in the concentration of deoxyhemoglobin, which occurs due to an increase in cerebral blood flow during brain activity. Most studies examining BOLD response function are based on the canonical hemodynamic response function (HRF, a measure of change in the magnetic resonance signal) on the premise that the HRF in stroke patients follows the same temporal sequence as normal individuals. However, this assumption is questionable as the fundamental issue with cerebrovascular infarction is the altered temporal dynamics of blood flow to the damaged hemisphere. From a practical standpoint, this translates to a misinterpretation of a lack of significant activation in brain regions in poststroke patients as indicative of absence of evidence of language recovery or reorganization, when in fact, the lack of activation is due to a measurement artifact. Not all studies, however, that have examined the HRF in individuals with poststroke aphasia have shown consistent findings. In one study, Bonakdarpour and colleagues [7] examined the time course of the HRF in patients and controls performing a simple lexical decision task. While the behavioral data showed that patients performed at high levels of accuracy, slowed HRF and time to peak in the left-perisylvian regions (relative to the visual cortex) was observed in three of out five individuals with chronic aphasia. In a followup study by the same group of researchers [20], patient-specific hemodynamic response functions were obtained on an fMRI task that utilized long interstimulus-interval trials. When these corrected HRF functions were taken in account for each of six patients who underwent a sentence production treatment, regions that showed greater activation after treatment were associated with shorter time to peak latencies (TTP) and increased perfusion. In another study, Peck and colleagues [21] examined three patients with aphasia and three controls on a language task before and after therapy. They also examined time to peak (TTP) in selected ROIs before and after therapy and found that improvements in therapy was associated with a decrease in TTP. In general, these studies suggest that reduced perfusion in a region results in a longer time to peak ratios even in chronic patients, and despite this apparent disadvantage, changes in activation in these regions and reduction in TTP latencies can be observed as a function of rehabilitative treatment.

While the previous studies examined the temporal dynamics of HRF in individuals with chronic poststroke aphasia, a few other studies have examined HRF timing in acute and subacute patients. In one study, Altamura and colleagues [22] examined BOLD HRF at two time points (acute and subacute) stages in six patients with aphasia and their normal controls. Results revealed that BOLD HRF had decreased amplitude and longer TTPs in the subacute stage but not the acute stage, indicating that as time progresses from the onset of the infarct, there is a deterioration of the cerebral hemodynamics in the impaired hemisphere.

These findings generally call in to question the validity of examining canonical HRF in individuals with poststroke aphasia as it is easy to underestimate the nature of activation by overlooking a temporally delayed HRF in regions with hypoperfusion. In contrast, van Oers et al. [23] used a different paradigm known as the breath-hold task to examine the nature of cerebral vasculature and found no differences in the hemodynamic responsiveness in the left and right hemisphere in 13 chronic patients and 13 normal controls. This study then also found activation in the left IFG in these chronic stroke patients for two language tasks indicating that in the presence of normal HRF, ipsilesional regions are activated in the service of language function.

Like studies on poststroke aphasia, studies on motor recovery following a stroke have also documented delayed HRF. For instance, Roc et al. [24] examined the temporal response of the HRF in a visuomotor task in 7 patients with significant stenosis in the ICA/MCA and 7 normal controls. Relative to controls, patients exhibited a delayed HRF response in the primary motor cortex despite the absence of any behavioral differences on the motor task. In another study, Pineiro et al. [25] examined the time course of the BOLD HRF in 12 patients with lacunar strokes on a motor task. Notably, results of this study found that latency and peak amplitude of the BOLD signal was lower in the patients relative to controls in the sensorimotor cortex in both the affected hemisphere as well as the unaffected hemisphere, indicating that altered BOLD HIRF time course may occur due to diffuse cerebrovascular pathology or due to preexisting pathophysiological changes in the cerebral microvasculature.

To summarize, while it is clear that there are changes in the time course of the BOLD HRF in chronic individuals with stroke and even in subacute individuals with stroke, the precise mechanisms of these changes are not well understood. On the one hand, studies that document altered HRF temporal dynamics in both hemispheres are indicative of varying differences in the cerebral microvasculature subsequent to the stroke that may influence both hemispheres. On the other hand, studies that document altered HRF only in the affected hemisphere claim this phenomenon to be an outcome of cerebrovascular abnormalities due to the infarct and coincide with hypoperfusion within peri-infarct regions. Importantly, the few studies that have documented the time course of the HRF have also shown it to improve as a function of rehabilitation. Future research will need to clearly delineate the nature and variability of temporal course of the HRF, its consequence on behavioral function, and its potential to change. 

### 2.2. What Is the Nature of the Peri-Infarct Tissue in the Patients with CVA?

Recall that the HRF is a measure of change in the magnetic resonance signal as a consequence of change in the blood's metabolic needs. Thus, measurement of HRF is directly related to the amount of perfusion (blood flow) in the brain tissue and consequently, the anatomical viability of the tissue in the peri-infarct cortex. This peri-infarct cortical tissue can range from the ischemic penumbra (defined as the area of moderately ischemic brain tissue surrounding an area of more severe ischemia) to regions that are slightly remote from the core of the lesion but is functionally/anatomically connected to the infarcted tissue. Specifically, ischemic penumbra is the area surrounding the core infarct that is getting enough blood to survive but not enough to function [26]. The ischemic threshold of cerebral perfusion for membrane failure is around 8 mL/100 g brain tissue/minute, in contrast to the normal blood flow of 20 mL/100 g/minute. Much research has been focused on addressing the issue of whether peri-infarct tissue is viable and if so, what role this tissue may play in poststroke recovery of language function. There are several current approaches to identifying and measuring the size of the ischemic penumbra [27]. One such approach is the calculation of the *diffusion-perfusion mismatch*. Diffusion-perfusion mismatch refers to the difference between volume of perfusion abnormality on perfusion-weighted imaging (PWI) and volume of diffusion abnormality on diffusion-weighted imaging (DWI) and allows the estimation of at-risk tissue and the potential for targeted intervention [28]. Work by Hillis and colleagues [29, 30] has shown that the degree of diffusion-perfusion mismatch in specific language regions that are hypo-perfused predicts the amount of improvement in language function during the acute stroke phase. Damage in these hypo-perfused regions can be reversed if blood flow can be elevated above anoxic values and is usually done by elevating the blood pressure with fluids or pressors, surgical intervention such as carotid endarterectomy, intra-arterial thrombosis, and internal carotid stenting. There are, however, several issues with the calculation of diffusion-perfusion mismatch, some of which include the overestimation of the ischemic penumbra [31].

Another approach to examining the peri-infarct tissue is by conducting arterial spin labeling perfusion (ASL) MRI to establish the cerebrovascular flow in tissues within regions of interest in the brain. Arterial spin labeling allows noninvasive absolute cerebral blood flow (CBF) measurements by using magnetically labeled arterial blood water which decays with the T1 relaxation rate as a diffusible flow tracer [32]. This technique is similar to positron emission tomography (PET) in that it measures temporal perfusion of blood into different regions of the brain, but PET uses radioactive oxygen, whereas ASL measures the magnetically labeled arterial water. Using ASL methodology, several studies have examined perfusion into the cortex as an index of the viability of tissue. For instance, Brumm et al. [33] examined CBF mean transit times to localize regions of hypoperfusion in three stroke survivors with chronic infarcts. Results showed abnormal transit delay times for the three patients and chronic hypoperfusion in the ischemic penumbra region. Interestingly, there was an inverse relationship with lesion size and ischemic penumbra ratio (*ratios* of average CBF between the lesion penumbra and each participant′s intact hemisphere) indicating reduced perfusion in a patient with a larger lesion than in the one with a smaller lesion.

In another study, Richardson and colleagues implemented ASL MRI in 17 left hemisphere stroke patients and found a decrease in perfusion in the peri-infarct tissue, and like Brumm et al., also reported that larger lesions were associated with decreased perfusion relative to smaller lesions [34]. In a followup study, Fridriksson and colleagues [35] examined the relationship between treatment outcomes and perfusion in perilesional tissues and found that changes in activation in perilesional areas especially in the left frontal lobe predicted improvements in naming and changes in the left temporal lobe was modulated by decreases in naming errors. Interestingly, an improvement in naming was predicted by baseline CBF values in the residual language network (brain regions typically involved in picture naming that were outside the perilesional regions). In both these studies, perilesional tissue was identified on the basis of stepwise effects of distance up to 15 mm from the edge of the lesion in the ASL scans that was averaged across patients. Hence, it is possible that the hypo-perfused tissue could extend beyond this region, indicating that hypo-perfused tissue in the peri-infarct region may be broader than just the ischemic penumbral region. In a related study on motor recovery, Krainik et al. [36] observed decreased BOLD signal amplitude in sensorimotor and supplementary motor cortices in ipsilesional hemispheres in chronic stroke participants during a manual motor task even when the neural regions were structurally intact.

To summarize, using different methodologies it is clear that peri-infarct tissue and particularly the ischemic penumbra remains hypo-perfused. But what the functional significance of hypoperfusion in the chronic stroke brain is remains unclear as both Fridriksson et al. and Krainik et al. have shown that activation in perilesional tissue is associated with improved behavioral outcome even though this tissue may be hypo-perfused. One way to resolve this debate will be to combine ASL and fMRI procedures to carefully examine the relationship between the extent of perfusion in the peri-infarct tissue, the extent of BOLD activation in that region and improvements in behavioral performance.

### 2.3. What Are the Neuronal Changes in the Peri-Infarct Tissue and Ipsilesional Hemisphere?

Independent of whether there is hypoperfusion in the peri-infarct tissue, an important aspect of physiological recovery after a stroke are the cellular changes that occur around the lesion that include an increase in dendritic spines, axonal sprouting, and consequent neurogenesis in the cortex adjacent to the infarct tissue [37–39]. In general, it appears that the ischemic infarct triggers a series of cascading cellular events that involve several neuroplastic changes in the peri-infarct cortex that ultimately predispose this region to participate in reorganization of function. Further, studies in adult rodent have shown an increase in neurogenesis to forebrain regions after a stroke. For instance, Kreuzberg and colleagues [40] found an increase in neuroblasts derived from the subventricular zone of the lateral ventricles in the ipsilesional hemisphere and peri-infarct zone 14 and 35 days after the onset of a stroke in adult rats. The authors suggested that these neuroblasts eventually form mature neurons indicating that neurogenesis can indeed occur in the peri-infarct zone in poststroke brains.

Related to the previous topic, change in dendritic spine density plays an important role and is potentially related to the extent of perfusion in the lesioned hemisphere. In one study, Mostany et al. examined dendritic spine turnover in regions adjacent and distant from the core of the infarct in mice and found that tissue perfusion influenced the nature of dendritic plasticity in the peri-infarct cortex. Specifically, blood flow to the peri-infarct region increased in a three month period but not to pre-stroke levels, and dendritic spines near the core of the infarct (and the lower ischemic regions) were maintained over time, whereas regions distant from the lesion that had improved blood flow showed an increase in dendritic spines over time. These authors suggested that restored blood flow in regions proximal and distal to the core of the infarct could influence the nature of dendritic plasticity in the ipsilesional hemisphere, but that plasticity may be greater in regions distant from the lesion than closer to the ischemic hemisphere.

These physiological and cellular changes in the peri-infarct tissue lend further credence to the observation that rehabilitative training in animals following stroke has been found to result in an improvement in the representational dendritic plasticity; specifically, regions surrounding and contralateral to the lesion that recruited to subserve function [41–46]. Recent studies examining motor rehabilitation in poststroke animals have shown several physiological events that occur ensuing rehabilitation/behavioral experience that include, among other things, attenuation of cell death, stimulation of dendritic growth, and increase in dendritic spine density [41, 47–49]. Further, targeted motor rehabilitation of the impaired limb in animals increases the survival of the residual tissue in the ipsilesional cortex [50]. These principles of plasticity are also true in motor recovery in human adults [16, 17], and thus have direct relevance for reorganization of language function in poststroke aphasia. To summarize, there may be altered temporal dynamics of the perfusion to the infarcted regions in the brain, and this may directly affect the way the BOLD response function is measured. While reduced perfusion to the infarcted cortex may be worse in regions proximal to the infarct relative to regions further away from the lesion, there are several physiological and cellular changes in the peri-infarct cortex, which despite the reduction in perfusion, may facilitate the residual tissue to be engaged in neural plasticity to support language recovery. Therefore, as long as there is evidence of structural, physiological, and functional integrity of the peri-infarct tissue, and the predisposing factors promoting neurogenesis are present, the prognosis for language recovery in the infarcted hemisphere appears favorable.

## 3. What Are the Patterns of Reorganization of Language?

One of the main advances in the field of neuroimaging of aphasia is the fairly large body of evidence documenting the reorganization and recovery of language function. In this next section, the nature and extent of recovery is discussed within each phase of recovery.

### 3.1. Acute Phase: Reperfusion of Tissue

Language recovery in the acute phase (typically in the first few weeks after the infarct) is mostly determined by the extent of successful reperfusion of the infarcted tissue in order to restore language function. For instance, in an early study, Hillis and Heilder [51] examined 18 patients with acute left hemisphere infarcts and resulting impaired spoken word comprehension abilities. Upon reperfusion to an area of hypoperfusion in the Wernicke's area (left posterior superior temporal gyrus), these patients improved in their word comprehension ability. (In this paper Wernicke's area refers to the posterior superior temporal gyrus.) Subsequently, in other studies, Hillis and colleagues have documented hypoperfusion and subsequent improvements in language function upon reperfusion in (a) Wernicke's area with a decrease in severity of semantic processing impairments [52], (b) regions in the middle temporal, fusiform, Broca's and Wernicke's area [1] and in improvement in naming function, and (c) LIFG and an improvement in comprehension and production of sentences and motor planning of speech articulation [53]. (In this paper Broca's area refers to the pars opercularis and pars triangularis portions of the IFG.) These studies illustrate that reperfusion of the hypo-perfused area during the acute stages is a critical component of recovery of language and also underscores of the importance of these regions in various aspects of language processing in the brain.

### 3.2. Subacute Phase: Resolution of Diaschisis

In most cases, reperfusion can only salvage the ischemic penumbra for the first few days following ischemia and eventually, the hypo-perfused area often progresses to infarction [31, 54, 55]. Nonetheless, language recovery continues to occur in the ensuing months following the stroke. In a seminal paper, Saur et al. [56] used repeated fMRI examinations with parallel language testing to examine the reorganization in the language system from the acute to the chronic stage in 14 patients with poststroke aphasia. The authors showed that brain reorganization during language recovery proceeded in three phases. In the initial few days after stroke, the activation of the left language areas was strongly reduced; whereas in the second phase around 12 days after stroke, there was an upregulation of the entire language network with recruitment of homologous language regions. By the end of a year, there was a normalization of fMRI activation with peak activation shifting to the left hemisphere and a decrease in the right hemisphere activation. This was an important study because it showed restoration of language function to the left hemisphere over time that corresponded with improvements in language function.

One of the mechanisms thought to be at play during subacute phase (three–six months after stroke) involves the resolution of diaschisis, that is, a decrease in hypometabolism of structurally normal regions remote from the infarct due to disruption of a functional pathway to those regions. Distant effects of pathophysiological reorganization occur either as reduced activation in remote regions (dynamic diaschisis) or increased activation of remote regions (disinhibition of functionally redundant systems). Several studies suggest that resolution of diaschisis results in the improvement of language functions in patients with aphasia in the subacute phase. For instance, Cappa et al. [57] examined eight patients with unilateral left hemisphere stroke using PET in the acute phase after stroke (within 2 weeks) and 6 months later. While all participants showed substantial behavioral recovery by six months, PET scans showed an increase in CBF in the six month scan compared to the first scan. The authors concluded that language recovery in the first months after aphasia is associated with regression of functional depression (diaschisis) in structurally unaffected regions, in particular in the right hemisphere.

In another study, Price et al. [58] investigated remote diaschisis using PET in four patients with aphasia six months after the onset of stroke, all of whom had lesions to the inferior frontal gyrus. While non-brain damaged participants typically showed activation in IFG, MTG, and posterior ITG, the patients showed decreased activation in the ITG (but not MTG) as this region was thought to depend upon inputs from the IFG which, in this case, was damaged.

Interestingly, Fair and colleagues [8] have a slightly different interpretation of remote diaschisis. In their study, they examined three patients who were scanned on a PET rCBF and a word-stem completion fMRI task. The authors found that even though rCBF measures were significantly lower in the lesioned hemisphere even in regions remote from the lesion relative to the intact hemisphere, these regions showed normal activation patterns on the fMRI task. This finding is consistent with the studies reviewed above examining the degree of perfusion in the peri-infarct cortex indicating that diaschisis and hypoperfusion are related. To summarize, studies in the subacute phase indicate that recovery of language is dependent on the persistence of hypoperfusion and hypometabolism in regions proximal and distant from the site of lesion. It is likely that the resolution of diaschisis is dependent on the extent of neural plasticity possible in the peri-infarct tissue and the nature and scope of behavioral rehabilitation.

### 3.3. Chronic Phase: The Role of the Ipsilesional Hemisphere

Finally, studies examining recovery of language in the chronic stage (>9 months) have addressed the issue of whether recovery of language function in the chronic phase is a simple reversal of normal left hemisphere lateralization (i.e., transferring language functions as a whole to the right hemisphere) or exclusive recruitment of left perilesional and other language areas [3–5] or a combination of the two. A recent meta-analytic review of 12 studies by Turkeltaub and colleagues [59] found control participants and patients with aphasia activated overlapping regions in the left frontal and temporal regions. Importantly, patients with aphasia showed activation in left hemisphere regions such as IFG and MTG that was also observed in control participants, new left hemisphere regions such as anterior insula and MFG that were not observed in control participants, and homologous right hemisphere regions (not observed in control participants) such as RIFG, right postcentral gyrus (RPCG), and RMTG. Turkeltaub et al. concluded that patients with limited damage to the dominant/left hemisphere may demonstrate improvements due to reengagement of spared regions and may also recruit alternate perilesional areas to subserve language recovery. In patients with large left hemisphere lesions, the engagement of the contralateral right hemisphere homologues, particularly the RIFG is crucial to successful recovery of language. To summarize, the results from this meta-analysis and other subsequent studies not included in the meta-analysis [23, 60] suggest that because left hemisphere is lateralized for language, engagement of the left hemisphere is crucial for behavioral improvement in aphasia.

While the review provided by Turkeltaub et al. is an important foundation for making predictions about the possible mechanisms underlying recovery, this review mostly includes studies that document spontaneous or natural language recovery when most of the observations are made at one or two time points during the course of recovery, thus, the impact of these findings to language reorganization after rehabilitation is not immediately apparent. Therefore, no conclusive inferences can be made about the specific contributions of activated neural regions when aphasic patients are scanned during a single experimental session irrespective of whether they are able to perform a behavioral task. As will be discussed below, longitudinal fMRI scans (with or without rehabilitation) are an important methodological approach to understand the nature of language reorganization in the brain.

The study of the neural basis of treatment induced language recovery in patients has been more informative at identifying regions of the brain that are engaged in the recovery of language [9, 61–66]. Again, there is some debate about whether improved behavioral responses during language processing tasks are associated with concomitant changes in the left perilesional regions or homologous right hemisphere regions. Several factors including lesion site/size, performance in the scanner, amount of behavioral improvement, and nature of the therapy protocol confound the resolution of this issue. Several studies have specifically targeted word retrieval in therapy. A study previously described by Peck and colleagues [21] studied the relationship between hemodynamic response (HDR) peaks in right hemisphere and response latencies in a word generation task in three patients who underwent anomia treatment. The results showed primarily right hemisphere changes in the temporal aspects of the HDR during the word generation task.

In another study, Vitali et al. [67] examined the neural correlates of improved picture naming performance in two patients with anomia using a phonological cueing treatment. Increased perilesional activation as a function of treatment was observed in one patient who showed no damage in the left IFG, whereas increased right IFG activation was observed in another patient with a large left hemisphere lesion. Davis et al. [68] reported a treatment that used semantic aspects of the target to increase selection of the target under competition. Treatment required selection of semantic attributes of each target item without overt naming of the object, and therefore the authors hypothesized that treatment should help inhibiting competitors in productive speech. Similarly, Crosson et al. [69] predicted that pairing a complex left hand movement task with naming should trigger a right medial frontal intentional mechanism but measured changes in the scanner using a picture naming task. The authors suggested that an increase in right Pre-SMA activity should also increase right lateral frontal activity and improve language (naming). Finally, Raboyeau et al. [66] trained 10 patients in a word learning paradigm that provided individuals with the image of the object, followed by the spoken first syllable and written first syllable and then the name of the object. PET scans were performed on a picture naming task, and results revealed increased activation in the right insular and right frontal regions as a function of treatment.

Work done by Fridriksson et al. [70, 71] suggest that both hemispheres can be recruited to support treatment-induced naming recovery in aphasia. In the first study, Fridriksson et al. [70] investigated changes after treatment focused on a combination of spaced retrieval training, errorless word learning, and massed practice that was provided in a loosely organized group treatment setting. A picture naming fMRI task conducted before and after treatment revealed a bilateral increase in neural activity associated with improved naming ability in two participants, while the third did not respond to treatment. In the followup study, Fridriksson et al. [71] investigated phonological and semantic-based treatments in three patients. Again two of the three patients exhibited a trend toward improved naming, whereas the third did not. Posttreatment fMRI once again revealed bilateral changes in activity, located not just in language-related cortex but also in regions outside of the traditional language regions. In contrast, in a recent study, Fridriksson [62] examined 26 patients with aphasia who received consecutive semantic and phonological cueing therapy to improve naming. Results revealed an increase in left hemisphere undamaged frontal and parietal regions in patients who demonstrated an improvement in naming skills after treatment.

The notion of the importance of left hemisphere perilesional activation as a function of improved picture naming skills after treatment has been espoused by other researchers also. For instance, Léger et al. [72] reported increased perilesional activation as a function of treatment in one patient who received a phonological based rhyme judgment treatment to improve naming skills. In a series of studies, Meinzer and colleagues examined the neural correlates of picture naming as a function of therapy. The nature of therapy provided varied across studies ranging from unspecified language treatment for one aphasic individual [73], constrained induced language therapy for 11 aphasic individuals [64], and a phonological/orthographic cueing hierarchy with eight aphasic individuals [65]. Of these, one study found increased activation in the RIFG subsequent to improved naming [73], one found increased perilesional activation associated with improved naming [64], and one found increased activation in the hippocampal regions related to success in therapy immediately after treatment and in the right temporal lobe approximately eight months after therapy [65].

While the studies reviewed above have mainly focused on recovery of word retrieval (one of the most common language deficits in poststroke aphasia), there are other studies that have examined recovery of sentence production and comprehension that mirror the findings observed in word retrieval. Specifically, while Cherney and Small [74] have found increased activation in the right hemisphere in their two patients, Thompson et al. [20] found bilateral activation in the temporoparietal regions in their six patients and Wierenga et al. [75] found increased activation in the LIFG after treatment in two patients.

To summarize, the evolution of studies examining rehabilitation in poststroke aphasia increasingly points towards the principal engagement of perilesional regions in supporting training induced language recovery and is consistent with Turkeltaub et al.'s [59] suggestions about the role of the left IFG and perilesional regions in natural language recovery. To reiterate this point, positive outcomes with therapy are associated with reengagement of the left hemisphere and perilesional regions. These findings are consonant with the observation made in the first section of this paper. When the peri-infarcted tissue in the infarcted hemisphere retains its structural and functional integrity, there is a strong likelihood of language to be reorganized to these regions. In cases when the right hemisphere is involved in language recovery, it is not very clear what the role may be; it may be compensatory, it may be engaged in the service of processing more difficult information, or incorrect information or it may provide a temporary/transient role in complementing the functions of the left hemisphere. Nonetheless, what is apparent is that reorganization of language does not include activation of one or two regions in the brain (albeit in the peri-infarct zone), rather there are large scale networks that are engaged in the service of plasticity.

## 4. What Are the Other Mechanisms of Change Documented in Language Recovery?

The fact that language recovery or reorganization in the brain involves several regions in the brain is not a surprising finding; especially given the unambiguous observation that even in normal individuals, language processing involves a network of regions. First, regions in the brain such as the IFG, MTG, or IPL engaged in language processing do not function as isolated modules; these regions are highly anatomically interconnected with each other and with other regions. Second, even regions that are not anatomically connected tend to function as temporally synchronous units suggesting a level of functional connectivity between regions engaged in the service of language processing and consequently language recovery.

### 4.1. White Matter Changes as a Function of Language Recovery

Several studies have combined BOLD signal diffusion tractography to understand language processing networks in normal non-brain damaged brains. One such study by Saur et al. [76] implemented a combination of diffusion tensor imaging (DTI) and an fMRI study using a word/nonword repetition task and a sentence/nonsentence comprehension task and identified two distinct fiber pathways for these language tasks. The first dorsal pathway comprising the arcuate fasciculus and superior longitudinal fasiculus connected the superior temporal lobe and the premotor cortex and was involved in the phonetic-motor mapping required for word/nonword repetition. The second ventral pathway comprised the extreme capsule (white matter fibers connecting the posterior aspect of Wernicke's area, claustrum, insula, and Broca's area) with connections from the MTG and the ventrolateral prefrontal cortex. In a related study, Turken and Dronkers [77] examined the connectivity of specific regions in the left hemisphere that included the posterior MTG, anterior STG, lateral STG (BA 22), orbital IFG (BA 47), MFG (BA 46), and AG (BA39). For each of these regions, the authors examined the structural and resting state connectivity to other regions in the brain and observed that the MTG was especially interconnected with other regions in the brain. Additionally, they also found that the arcuate fasciculus, inferior occipitofrontal and middle and inferior longitudinal fasciculi, and the uncinate white matter fiber pathways were structurally connected to the regions of interest defined above and connected to other language regions in the brain. In contrast, Morgan et al. [78], using a similar methodology of combining an fMRI resting state connectivity with DTI measures, found that there was more variability across normal brains in terms of the structural and functional relationship between language areas of the brain. Thus, while there was a relationship between mean radius bundle of the white matter fibers and resting state connectivity between Broca's area and the supplementary motor area, there was no clear relationship between fractional anisotropy and resting state connectivity for the connection between these two regions. Similarly, there were multiple pathways between Broca's area and Wernicke's area that highlighted the potential variability across subjects.

In addition to connectivity between regions in the language networks, several recent studies have examined connectivity between specific regions within the left hemisphere. For instance, Kaplan et al. [79] examined the structural connectivity of the horizontal fibers of the arcuate fasciculus to pars triangularis and pars opercularis in the IFG and the ventral premotor cortex in the two hemispheres and found that connections from the horizontal fibers of arcuate fasciculus to pars opercularis were more consistent than connections to pars triangularis in normal individuals. There was, however, a slight hemisphere preference across the normal individuals. Specifically, in the eight normal subjects they examined, all eight showed these connections between arcuate fasciculus and pars opercularis in the left hemisphere, whereas only five individuals showed the same connections in the right hemisphere. Such findings indicate that there is a certain amount of variability in structural connectivity even in normal brains, which obviously has implications for the way reorganization of language is interpreted. In another study, de Zubicaray et al. [80] have examined the structural connectivity of the anterior temporal lobes, a region often implicated in the processing of semantic memory. Consistent with the findings of Turken and Dronkers, de Zubicaray et al. found that the inferior occipitofrontal longitudinal fibers and the uncinate fibers were important connectors between regions in the anterior temporal lobe, posterior temporal, and posterior/inferior parietal lobes. 

To summarize, in normal individuals networks of gray matter regions including IFG (pars triangularis, pars opercularis), MTG, STG, STS, and MFG are connected through the arcuate fasciculus, extreme capsule, uncinate fasciculus, and the occipitofrontal longitudinal fasciculus. Understanding the normal structural connectivity is extremely relevant to understanding recovery of language in poststroke aphasia as presumably, recovery progresses along the reestablishment of normal structural connections within the language network.

A few studies have examined changes to white matter connections in individuals with poststroke aphasia. For instance, Breier et al. [81] examined behavioral and neural changes after constrained induced language therapy (CILT) in one individual with aphasia. Behavioral improvements subsequent to treatment were accompanied by bilateral changes in the temporal lobe, inferior frontal lobe/insula, STG, MTG, SMG, and AG as measured by magneto encephalography (MEG) experiments, as well as an increase in the integrity of the arcuate fasciculus bilaterally (as measured by DTI).

In another study, Schlaug et al. [82] also examined the integrity of the arcuate fasciculus and subsequent treatment related changes in six individuals with aphasia who received melodic intonation therapy (MIT). Schlaug et al. found an increase in the number of AF fibers and volume that correlated with behavioral changes (in percent information content units provided) as a function of treatment. These results were taken to indicate that a therapy like MIT resulted in greater connectivity between the temporal lobe and frontal lobe thereby improving the integrity of the AF fibers. Therefore, studies on white matter changes are only beginning to inform us about structural plasticity of white matter fibers in language recovery subsequent to rehabilitation in poststroke aphasia. In contrast, there have been several studies that have documented white matter changes associated with motor rehabilitation in stroke patients [83] and have generally provided consonant findings to that of language recovery studies. Another paper by the Schlaug group [84] examined 15 patients who each received a combination of physical/occupational therapy and transcranial direct current stimulation and underwent a DTI scan to examine the integrity of specific white matter regions (ipsilesional pyramidal tract, ipsilesional descending corticospinal tract, and transcallosal motor tracts). Results showed positive correlations between tract specific diffusivity and change on a behavioral motor task with the transcallosal motor fibers emerging as the best predictor of change in motor function. In another related study, Qiu et al. [85] further found that white matter FA asymmetry in posterior limb of the internal capsule was better associated with behavioral motor function than a task related BOLD signal. What the Lindenberg study and other studies [86] point out is that the closer the structural integrity of specific white matter tracts is to normal non-damaged brains, the higher the functional outcome in the stroke patients. In addition to examining white matter connectivity between regions in the brain, a number of studies have also examined functional connectivity between regions of the brain.

### 4.2. Functional Connectivity and Rehabilitation Studies

In general, measures of functional connectivity examine regions of the brain that are statistically synchronous based on a specific task. The main aspect of functional connectivity is that the relationship between specific regions is time dependent. Analyses of changes in functional connectivity—often referred to as effective connectivity analyses—allow researchers to make inferences not only about the coupling among brain regions, but also about how that coupling is influenced by changes in the experimental context and the direction of the effect. The two main methods of exploring changes in functional connectivity are dynamic causal modeling (DCM) and structural equation modeling (SEM), both of which are routinely used with fMRI data. Both methods allow researchers to determine the influence of regions of interest on each other and also the influence of the experimental tasks on the connection strengths between these regions. DCM uses Bayesian estimation while SEM uses correlation and regression to determine these relationships.

A few studies have implemented this methodology to examine reorganization of language in poststroke aphasia. Abutalebi et al. [6] used DCM to examine the effect of treatment in one bilingual patient with aphasia on two different networks: the control network and the language network. The connections in these networks were measured for both languages (L1 = native language, L2 = second language) before and after therapy in L2. The authors found that treatment in L2 strengthened connections within the L2 language network, but weakened connections within the L1 language network. The reverse was true for selected connections in the control network. These subtle changes in the two networks for each language were not apparent with traditional fMRI analysis alone. Although this is a case study primarily examining changes in a bilingual language network, it highlights the utility of effective connectivity analysis in teasing apart specific effects of therapy at the single-subject level.

In another study, Vitali et al. [87] used SEM to explore treatment-induced changes in connectivity among four left hemisphere language areas (IFG, MTG, insula, and IPL) and their right hemisphere homologues in two patients. Both patients showed behavioral improvements due to treatment, and these improvements were associated with increases in connection strengths. The particular connections that were strengthened depended on if the items were trained or untrained and varied between the two patients depending on lesion characteristics. For both patients, more connections that were strengthened appeared during trained items than during untrained items. Also, the patient with the larger lesion had more connections strengthened in the right hemisphere, whereas the patient with the smaller lesion had more connections strengthened in the left hemisphere. While this is a somewhat preliminary study, examining functional connectivity at the single-subject level is important from a methodological standpoint. 

Finally, Sarasso et al. [88] compared left hemisphere and right hemisphere networks in four patients to a normative model at several time points throughout a treatment to improve articulation. They found that as treatment progressed, patients' left hemisphere networks (using structural equation models) more closely resembled (i.e., were a better fit to) that of the normative model, whereas right hemisphere networks started out resembling the normal network, but progressively resembled it less. Similar to the previous studies, one drawback of this study is the small number of participants. However, these results are interesting because they show a progressive shift in network connections throughout treatment. Where traditional activation maps may not be sensitive enough to show a gradual progression of treatment-induced reorganization, effective connectivity analysis was able to provide information about these subtle differences. Sarasso et al.'s study is also notable in that it provides further evidence about the role of the right hemisphere in the recovery of aphasia, in that it suggests that the optimal reorganization pattern for patients is to become more normal-like in the course of language rehabilitation. On the other hand, it is interesting to note that even in normal individuals, there is a certain amount of variability inherent in the functional connectivity. For instance, Veroude et al. [89] have shown that resting state functional connectivity is distinctively different in individuals who ultimately end up successfully learning a new language versus those who do not successfully learn a language. Specifically, in this study they found different resting state connectivity within regions involved in phonological processing in good learners of a Chinese weather forecast compared to the same regions in the brains of individuals who were not good learners. Therefore, there is inherent variability even across normal individuals that predisposes certain individuals to learn language more effectively than others. Consequently, the gold standard for measuring reorganization of language may not be “normal” language performance but by comparing each individual's own longitudinal language processing abilities.

### 4.3. Resting State and Functional Connectivity in the Default Mode Network

Complimentary to the analysis of functional connectivity, several studies have examined the nature of resting state networks devoid of any task-related activation and thus provide an index of temporal coherence between different brain regions. Also referred to as the default mode network (or task negative network), regions of the brain that are involved in “internal state processing” include medial temporal lobe (MTL), medial prefrontal cortex (MPFC), inferior parietal cortex, ventral precuneus, and the posterior cingulate cortex. There are, however, studies that have examined domain-specific resting state networks including sensorimotor, language, and visual processing. Two important papers have established the relationship between resting state connectivity and structural connectivity. One of the papers [77], already discussed above, examined the connectivity between the MTG and other language-related regions in the brain. The other study, by Greicius et al. [90] showed that regions within the default mode network including the MTL and MPFC and the posterior cingulate cortex are structurally connected.

There are no studies to date that have examined the nature of a disrupted resting state connectivity in individuals with poststroke aphasia. There are, however, a few studies that have examined the effect of disruption within resting state motor networks on motor impairment and recovery [91] for a comprehensive review. Three studies are discussed here as they are particularly relevant and can be translated to studies examining resting state connectivity in poststroke aphasia. In one study, Carter and colleagues [92] examined 23 stroke patients with motor and attentional deficits who also performed a resting state fMRI task (rsfMRI). Results of the functional connectivity analysis showed a reduction in connectivity between interhemispheric homologous sensorimotor regions that was significantly correlated with the extent of motor deficits and was a good predictor of motor performance. This study also examined intrahemispheric connectivity in both hemispheres which was not well correlated with motor or attention performance. In another study, Park et al. [93] examined motor networks in a longitudinal fashion, with multiple resting state fMRI sessions collected over a period of six months in 12 poststroke patients. Results showed that ipsilesional connectivity in the subcortical sensori-motor network between regions in the cerebellum and thalamus increased in the acute phase of the stroke. Also, the laterality index was higher (more ipsilesional connectivity) during the early parts of recovery (at onset and after one month), whereas connectivity became more symmetrical as time progressed. A third study examined changes in resting state connectivity subsequent to an accelerated and functional upper limb motor rehabilitation program in five poststroke patients [94]. Results showed that after therapy, all patients showed increased connectivity from the ipsilesional premotor cortex to the contralesional premotor cortex, with the magnitude of connectivity corresponding to behavioral improvements. Additionally, four of the five patients showed increased intrahemispheric connectivity between the ipsilesional premotor cortex and the primary motor cortex as a function of treatment. To summarize, all three studies highlight the nature of dynamic changes in connectivity during the period early recovery after stroke as well as a function of rehabilitation. While the studies differ on their findings of whether changes in interhemispheric or intrahemispheric connectivity are ultimately associated with positive behavioral outcome, the different methodologies in each of these studies do not merit a direct comparison. Nonetheless, a common theme of these studies which is translatable to studies of language recovery in poststroke aphasia is the longitudinal comparison of changes and the utility of measuring resting state networks as “functional baseline” for measuring task-related activation changes consequently.

## 5. Conclusions

This paper focused on three main topics. The first topic pertained to the nature of anatomical and physiological substrates in the infarcted hemisphere in poststroke aphasia, including the nature of the hemodynamic response in patients with poststroke aphasia, the nature of the peri-infarct tissue, and the neuronal plasticity potential in the infarcted hemisphere. Studies that have examined these variables have mainly documented that the temporal dynamics of the BOLD HRF may be altered in the ipsilesional hemisphere, but this tissue is engaged during language processing. Further, it appears that even though the region around the tissue may be hypo-perfused, it is also a bed for several cellular neuroplastic changes that take place over time and likely a prognostic indicator of favorable language recovery. The second section of the paper reviewed the current neuroimaging evidence for language recovery in the acute, subacute, and chronic stages of recovery. Evidence from a large body of neuroimaging studies suggests that in the early stages after the infarct, reversal of tissue necrosis has positive implications for language functions to return to the left hemisphere; in the chronic stages, engagement of residual tissue in the infarcted hemisphere is again associated with good overall language recovery. These regions can be perilesional residual tissue, perilesional regions that are anatomically proximal but functionally different regions (e.g., SFG activation following an IFG lesion), or distant regions that are functionally connected to the lesioned region.

The third and final section examined changes in connectivity as a function of recovery in poststroke aphasia, specifically in terms of changes in white matter connectivity, changes in functional effective connectivity, and changes in resting state connectivity in poststroke individuals. First, it seems like some regions, such as the IFG and MTG, are important “hub” regions which are highly connected to other regions in the brain. Thus, structural integrity between these potential “hubs” and other regions determines the extent to which anatomically proximal and distant regions may be engaged in the service of language recovery. Next, the functional connectivity between regions normally working in tandem determines the likelihood of these regions also being engaged after stroke and in recovery. One question remains, however. Does recovery of language look more like a normal network or more like a reorganized network? Additionally, if there is individual variability in the normal language network, what does an individual's reorganized normal network look like? These are all unanswered questions, but ultimately important questions to address in order to target effective rehabilitation in individuals with poststroke aphasia. Notably, findings from studies examining language reorganization after rehabilitation have reported increased activation as a function of treatment. Studies, however, that have examined skill acquisition have also demonstrated a decrease in activation as a function of increased proficiency. This is also true in individuals learning a second language, where increased proficiency is associated with a decrease in activation [95]. Thus, future studies will need to examine whether reorganization of language in poststroke aphasia corresponds to a tighter, more coherent and efficient network of residual, and new regions in the brain. Also, further work needs to be done to examine if structural and functional integrity of language networks undergo changes in the first few months after stroke, thereby determining the fate of potential regions that may be involved. Answering these questions will go a long way towards being able to predict which patients are likely to recover language (almost) completely and which patients are likely to may benefit from future rehabilitation.
